# Mg-Based Micromotors with Motion Responsive to Dual Stimuli

**DOI:** 10.34133/2020/6213981

**Published:** 2020-08-04

**Authors:** Kang Xiong, Leilei Xu, Jinwei Lin, Fangzhi Mou, Jianguo Guan

**Affiliations:** State Key Laboratory of Advanced Technology for Materials Synthesis and Processing, International School of Materials Science and Engineering, Wuhan University of Technology, Wuhan 430070, China

## Abstract

Mg-based micromotors have emerged as an extremely attractive artificial micro/nanodevice, but suffered from uncontrollable propulsion and limited motion lifetime, restricting the fulfillment of complex tasks. Here, we have demonstrated Mg-based micromotors composed of Mg microspheres asymmetrically coated with Pt and temperature-sensitive poly(N-isopropylacrylamide) (PNIPAM) hydrogel layers in sequence. They can implement different motion behaviors stemming from the driving mechanism transformation when encountering catalyzed substrates such as H_2_O_2_ and respond to both H_2_O_2_ concentration and temperature in aqueous environment. The as-constructed Mg-based micromotors are self-propelled by Pt-catalyzed H_2_O_2_ decomposition following the self-consuming Mg-H_2_O reaction. In this case, they could further generate bilateral bubbles and thus demonstrate unique self-limitation motion like hovering when the phase transformation of PNIPAM is triggered by decreasing temperature or when the H_2_O_2_ concentration after permeating across the PNIPAM hydrogel layer is high enough to facilitate bubble nucleation. Our work for the first time provides a stimuli-induced “hovering” strategy for self-propelled micromotors, which endows Mg-based micromotors with an intelligent response to the surroundings besides the significant extension of their motion lifetime.

## 1. Introduction

Inspired by microorganisms in nature, artificial micromotors that propel themselves by converting diverse energies have been designed to perform complex tasks, such as cargo transport [1–3], advanced nanomanufacturing [4, 5], and environment remediation in microscopic environments [6–11]. Their motile behavior also further endows them great advantages as next-generation nanorobotics used for biomedicine [12, 13].

Mg-based micromotors, as a typical class of self-consuming artificial micromotors, not only could be powerfully driven by the Mg-H_2_O reaction in physiological condition but also produce a byproduct of Mg^2+^ ions, an additional nutritional ingredient that benefits the human bodies [14, 15]. Additionally, the vast quantities of therapeutic hydrogen generated in the propulsion can further be used to eliminate excessive hydroxyl radicals which are harmful to living bodies [16]. They displayed superior compatibility for biological systems and have already been applied for drug delivery in the gastrointestinal tract of mice with enhanced therapeutic efficacies [17–22]. However, they generally suffer from poor motion controllability and relatively short motion lifetime stemming from the single driving reaction and self-consuming feature and cannot stop their movement unless the solid “fuel” Mg cores are almost consumed [23, 24]. To prolong the lifetime and improve the motion controllability of Mg-based micromotors, several strategies have so far been developed including tuning the opening size, controlling the pitting anions, and being functionalized with photocatalysts [21, 25], soft hydrogels [26], or biological materials [19, 25, 27, 28]. Yet they still face challenges in stimuli-responding propulsion and desirable “on/off” characteristics, and further efforts are necessary toward intelligence and controllability, which may lay the foundation for Mg-based micromotors to fulfill tasks in complex environments.

Here, we present a Mg-based micromotor with motion responsive to both H_2_O_2_ concentration and temperature in the surroundings. It consists of a Mg microsphere asymmetrically coated with Pt and temperature-sensitive poly(N-isopropylacrylamide) (PNIPAM) hydrogel layers in sequence. It can demonstrate the propulsion mechanism transformation between Mg-H_2_O reaction and Pt-catalyzed H_2_O_2_ decomposition and unique concentration- or temperature-induced self-limitation behaviors like hovering. The temperature-induced volume phase transformation (swelling/shrinking) of PNIPAM and the H_2_O_2_ concentration both affect the permeability of H_2_O_2_ into the hydrogel layer and thus make the generation of O_2_ bubbles in either single or bilateral sides, resulting in self-limitation motion. This work presents a stimuli-induced “hovering” strategy for the manipulation of Mg-based micromotors as well as a method for prolonging their lifetime by taking use of fuels from the surroundings. Given the possible changes of H_2_O_2_ concentration and temperature in human bodies, especially in diseased sites, the herein reported dual stimuli-responsiveness of Mg-based micromotors with extended motion lifetime may to some extent promise potential advanced biomedical applications.

## 2. Results and Discussion

**Figure 1:**
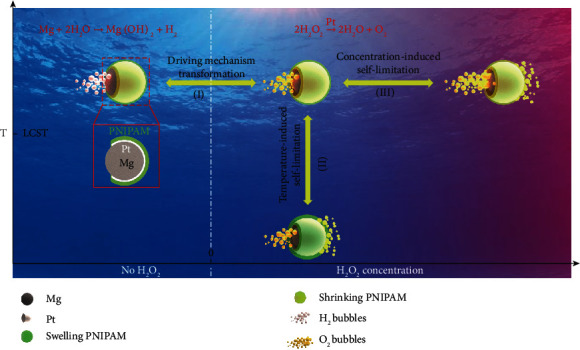
Schematic illustration of the Mg-based micromotor showing motion responsiveness to dual stimuli of temperature and H_2_O_2_ concentration. (I) The driving mechanism of the Mg-based micromotor is transformed from self-consuming Mg-H_2_O reaction to Pt-catalyzed H_2_O_2_ decomposition once H_2_O_2_ appears in the solution. (II) Temperature-induced volume phase transformation of PNIPAM induces the transformation of motion behaviors. When temperature is lower than LCST, the covered PNIPAM hydrogel layer swells to increase the amount of permeated H_2_O_2_ and thus the catalytically generated oxygen on the side of the PNIPAM hydrogel layer, resulting in a self-limitation motion of the Mg-based micromotor like hovering due to bubble O_2_ recoils from bilateral sides. (III) Concentration gradient of H_2_O_2_ induces the transformation of motion behaviors. Increasing H_2_O_2_ concentration raises the H_2_O_2_ concentration gradient between the two sides of the hydrogel layer, which facilitates H_2_O_2_ to cross the PNIPAM hydrogel layer and react with the middle Pt layer to form O_2_ bubbles. Consequently, a self-limitation behavior of the Mg-based micromotor occurs due to bilateral O_2_ bubble generation.

Given the motion environment diversity of micromotors when used, we focus on the changes of chemical reaction propulsion mechanism and dual stimuli-responsive motion behaviors of Mg-based micromotors in the liquid environment system. The aim is to achieve their long-term lifetime and motion manipulation. In view of the popularity of temperature difference and low concentration H_2_O_2_ in either biological or environmental system, we have integrated Mg microspheres with catalytically active Pt to H_2_O_2_ and temperature-responsive poly(N-isopropylacrylamide) (PNIPAM) hydrogels to construct Mg-based micromotors. They were fabricated by asymmetrically coating Mg microspheres with Pt and PNIPAM hydrogel layers in sequence via magnetron sputtering and UV polymerization techniques according to our previous literature [29]. The as-fabricated Mg-based micromotors are about 40 *μ*m in size and exhibit “opening” of about 14 *μ*m of the surface (Figures S1 and S2). As shown in Figure 1, the Mg-based micromotor when operated in an aqueous solution (e.g., containing NaHCO_3_ and poly(vinylpyrrolidone) (PVP)) is driven by asymmetrical H_2_ bubble release generated by Mg-H_2_O reaction due to the effective shielding of Pt layer from water in a gradually increasing confined space. When encountering catalyzed substrates such as H_2_O_2_, the propulsion mechanism of the Mg-based micromotor will transform spontaneously from the self-consuming Mg-H_2_O reaction into Pt-catalyzed H_2_O_2_ decomposition. As H_2_O_2_ (HO_2_^−^ + H_2_O + 2e^−^⟶3OH^−^, *E*^0^ = 0.878 V vs. SHE) has a stronger oxidization ability than H_2_O (2H_2_O + 2e^−^⟶H_2_ + 2OH^−^, *E*^0^ = −0.83 V vs. SHE) [30], the reaction of Mg and H_2_O_2_ is much easier to occur than the Mg-H_2_O reaction. As a result, O_2_ bubbles from Pt-catalyzed H_2_O_2_ decomposition instead of H_2_ bubbles propel the micromotor and the motion lifetime could be prolonged due to the transformation of driving reactions from the self-consuming mode to the catalytic mode. When the temperature is below the lower critical solution temperature (LCST, 32°C), the PNIPAM hydrogel is in the hydrophilic swelling state and has a large diffusion constant to solution [31]. Consequently, H_2_O_2_ aqueous solution can easily penetrate the gel layer to react with the middle Pt layer resulting in a temperature-induced self-limitation motion like hovering due to bubble O_2_ recoils from bilateral sides. Here, the self-limitation motion behavior means that a micromotor moves in 20 s only within a circle with the radius less than its twice size. The area of the motion region is characterized as *S*_20_ = *π*{(*x*_Max_ − *x*_0_)^2^ + (*y*_Max_ − *y*_0_)^2^}; here (*x*_Max_, *y*_Max_) is the farthest track point and (*x*_0_, *y*_0_) is the initial track point in 20 s. A similar transformation of motion behavior can also be triggered by increasing H_2_O_2_ concentration denoted as a concentration-induced self-limitation. A high concentration of H_2_O_2_ increases the concentration gradient between the two sides of the gel layer, inducing more H_2_O_2_ through the PNIPAM hydrogel layer for bilateral O_2_ bubble generation. In a word, the as-constructed Mg-based micromotor can implement different motion behaviors stemming from the propulsion mechanism transformation when encountering Pt-catalyzed substrates such as H_2_O_2_ and respond to both the concentration of catalyzed substrates and temperature in aqueous environment with extended motion lifetime. This suggests that the as-prepared Mg-based micromotor may be taken as an example to address its intrinsic drawbacks of uncontrollable propulsion and limited motion lifetime arising from its self-consuming features. Although the H_2_O_2_ concentration used here is not in the nontoxic physiological range of less about 50 *μ*M, the “fuel” concentration for the effective motor driving can be reduced via enhancing catalytic reaction activities, regulating motor structures, and so on [32, 33]. Moreover, the LCST of the hydrogel can be adjusted to fit the body temperature range by copolymerization [34, 35]. Thus, this will to some extent promote the development of Mg-based micromotors toward biomedical application.

**Figure 2:**
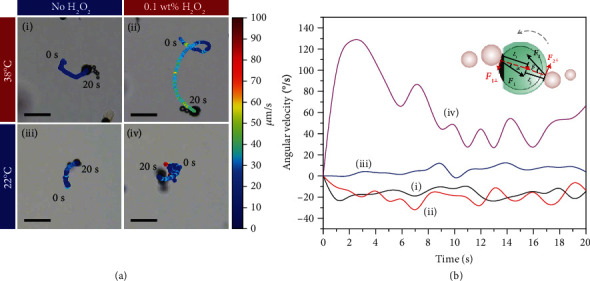
The motion responsiveness of the as-prepared Mg-based micromotors to temperature and H_2_O_2_. (a) Time-lapse microscope images of the Mg-based micromotor moving in aqueous solutions. Scale bar: 100 *μ*m. All systems contain 0.5 M NaHCO_3_ and 5 wt% PVP, and the color of the trajectory points represents the instantaneous speed of the Mg-based micromotor. (b) The corresponding angular velocities of the Mg-based micromotor in the four motion states of (a). The clockwise is specified as the positive direction. The inset is the schematic of force analysis for the Mg-based micromotor. The bubble breaks out of the micromotor and gives it a recoil (*F*_1_ and *F*_2_) that contributes forces (*F*_1⊥_ and *F*_2⊥_) for the motor rotation and translational motion toward the opposite direction of bubble generation, respectively. *α* and *β* are the angles between the directions of the centroid connection line and *F*_⊥_; *L*_1_ and *L*_2_ are the vertical distances between the center of mass of the Mg-based micromotor and *F*_1⊥_ and *F*_2⊥_.

Figure 2 and Video S1 show the motion behaviors of the Mg-based micromotor in the aqueous solution without or with 0.1 wt% H_2_O_2_ at 38°C or 22°C, respectively. All systems contain 0.5 M NaHCO_3_ and 5 wt% PVP to peel off the Mg(OH)_2_ passivation layer in Mg-H_2_O reaction and to facilitate the suspension of the micromotor [36–38]. As shown in Figure 2(a), the Mg-based micromotor displays an obviously increased speed from 14.3 *μ*m/s to 28.7 *μ*m/s when moving from the aqueous solution without H_2_O_2_ (Figure 2(a), i) to that with 0.1 wt% H_2_O_2_ (Figure 2(a), ii) at 38°C. The speed increase is caused by evidently accelerating bubble formation and detachment, suggesting the possible driving mechanism transformation from Mg-H_2_O reaction to Pt-catalyzed H_2_O_2_ decomposition. In the presence of H_2_O_2_, metal Mg prefers to react with H_2_O_2_ without H_2_ production rather than with H_2_O because of the higher reduction potential of H_2_O_2_ (0.878 V vs. SHE) than H_2_O (-0.83 V vs. SHE) [30]. Simultaneously, Pt-catalyzed H_2_O_2_ decomposition has a much higher rate constant (about 3.60 × 10^−2^ min^−1^) than Mg-H_2_O reaction (about 1.05 × 10^−2^ min^−1^) at an approximate pH of 7 [39, 40]. Additionally, the lower critical nucleation concentration of O_2_ (68 mM) is much lower than that of H_2_ (about 250 mM) bubbles [41, 42]. Both can induce vast quantities of O_2_ bubble generation to powerfully drive the motor.

As the PNIPAM hydrogel is a temperature-responsive polymer with LCST, it usually exhibits a sharp volume phase transition at LCST, which is slowed by further increasing/decreasing temperature [43]. On the basis of the swelling-shrinking kinetics of the PNIPAM hydrogel, the collective diffusion coefficient for swelling (*D*_0_ = 2.0 × 10^−7^ cm^2^ s^−1^) is about twice than that for shrinking (*D*_0_ = 1.1 × 10^−7^ cm^2^ s^−1^) [44, 45]. Although at the temperature below the LCST (32°C) the PNIPAM has a volume swelling with a large cooperative (collective) diffusion of the gel network [31, 44], the micromotor can only be propelled by asymmetrical bubble recoils during the Mg-H_2_O reaction because of the effective blocking from water by the middle Pt layer (Figure 2(a), i and iii). This will change when H_2_O_2_ appears in the system. As shown in Figure 2(a), iv, the micromotor shows an enhanced rotation with a decreased speed of about 15.8 *μ*m/s and a decreased *S*_20_, which looks like hovering because of bilateral bubble generation and release. This can be explained by the assumption that H_2_O_2_ aqueous solution may diffuse across the swelling hydrogel layer, reacting with the middle Pt layer to induce a self-limitation motion behavior of the Mg-based micromotor.

In order to explicate the temperature-induced self-limitation behavior, the corresponding angular velocity curves of Figure 2(a) are given in Figure 2(b). The single-side bubble-propelled micromotors (i, ii, and iii) show similar angular velocity curves with placid angular changes, while the bilateral-side bubble-propelled micromotor shows a relatively large angular change (iv). This can well explain the originality of the observed self-limitation state of the micromotor shown in Figure 2(a), iv. Under low Reynolds number conditions, the inertial effect can be neglected. The motion of the as-prepared Mg-based micromotor can be described by a set of differential equations for translation and rotation [46–48]:

(1) $$\begin{array}{c} m\frac{dv}{dt}=F_{\text{Stokes}}+F_{\text{Brownian}}+F_{\text{drive}},\\ J\frac{d\omega }{dt}=T_{\text{Stokes}}+T_{\text{Brownian}}+T_{\text{drive}}, \end{array}$$

where *m* represents the mass of the motor, *J* is the inertia tensor, and *v* and *ω* denote the translation velocity and angular velocity. The terms that distinguish these Langevin equations from standard Langevin equations for inactive Brownian particles are the driving force and torque contributions arising from bubble detachment. Thereinto, *F*_Brownian_ and *T*_Brownian_ represent the diffusion arising from the Brownian force and torque with zero mean. The purely Brownian force for a passive particle can be described by a theoretical translational diffusion coefficient (*D*_*T*_ = *k*_*B*_*T*/6*πηR*) [49] and a rotational diffusion coefficient (*τ*_*R*_^−1^ = *k*_*B*_*T*/8*πηR*^3^) [50]. In this system, as a spherical particle with a radius about 40 *μ*m, the characteristic time scale for rotational diffusion *τ*_*R*_ and translational diffusion coefficient *D*_*T*_ is calculated to be 728.5 min and 1.22 × 10^−10^ cm^2^/s, respectively. This means the effect of Brownian force on the motion of the as-prepared micromotor is limited. *F*_drive_ and *T*_drive_ are the reaction force and torque caused by the detachment of the bubbles, which will be balanced by the Stokes viscous drag force generated in the opposite direction during motion. Because of the spherical structure of the as-prepared micromotor, *F*_Stokes_ = 6*πηRv* (for translation) and *T*_Stokes_ = 8*πηR*^3^*ω*, where *R* is the particle (hydrodynamic) radius and *η* is the fluid viscosity. For the motion of the as-prepared Mg-based micromotor, the contributions of *F*_Stokes_ and *T*_Stokes_ are passive depending on *v* and *ω* and the rotation of bubble recoil-propelled micromotors mainly arises from the bubble detachment deviating from their symmetry [26, 51–53]. Therefore, a simplified force analysis of a micromotor based on the driving force is used to understand the motion states. *F*_1_ and *F*_2_ represent the instant propulsion forces provided by the bubble detachment from the opening and the coated side, respectively. For the cases shown in Figure 2(a), i, ii, and iii, *F*_2_ = 0 and the micromotor is only driven by single-side bubble recoil on the opening. Their rotational motions are all caused by the rotational component of *F*_1_ contributing an instant torque expressed as *T*_1_ = *F*_1⊥_*L*_1_ = *F*_1_*L*_1_ sin *α*, where *F*_1⊥_ represents the vertical component of *F*_1_ for motor rotation, *L*_1_ is the effective lever arm, and *α* is the angle between the directions of the centroid connection line and *F*_1⊥_. The differences in their motion states including translational and rotational velocities as well as rotational directions mainly depend on *α* and the bubble detachment positions. For the micromotor moving in 0.1 wt% H_2_O_2_ aqueous solution at 22°C, neither *F*_1_ nor *F*_2_ is 0 due to bilateral bubble generation and ejection. As a result, the instantaneous torque that contributed to the micromotor's rotation is the vector sum of the two torques: *T*_1_ (*T*_1_ = *F*_1_*L*_1_ sin *α*) generated by bubble escape from the open end and *T*_2_ (*T*_2_ = *F*_2_*L*_2_ sin *β*) generated by bubble release from the coating side. When *αβ* > 0, the micromotor exhibits an enhanced rotation corresponding to a large angular velocity (Figure 2(b), iv), while it shows a weakened rotation as *αβ* < 0 (Figure S3, Video S2). The self-limitation behavior of the Mg-based micromotor in the H_2_O_2_ aqueous solution below the LCST of PNIPAM is attributed to the enhanced rotation caused by the bilateral-side bubble detachments.

**Figure 3:**
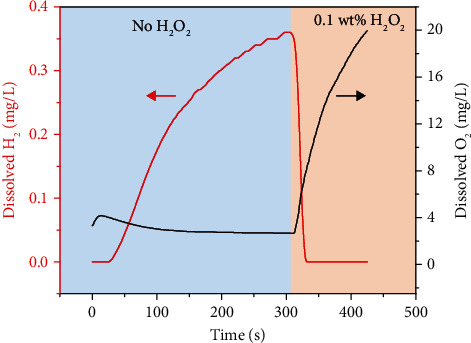
The diagram of dissolved H_2_ and O_2_ over time before and after adding H_2_O_2_ in the motion system containing 0.5 M NaHCO_3_ and 5 wt% PVP.

The dissolved H_2_ and O_2_ in the aqueous solution before and after adding H_2_O_2_ are determined to verify the driving mechanism transformation from the self-consuming Mg-H_2_O reaction to the Pt-catalyzed H_2_O_2_ decomposition.  Figure 3 shows that when the motors move in the aqueous solution without H_2_O_2_, the dissolved H_2_ in the solution increases gradually. This indicates that the driving reaction at this moment is a H_2_-produced reaction as Mg + H_2_O + 2HCO_3_^−^⇌MgCO_3_ + CO_3_^2−^ + 2H_2_. When H_2_O_2_ is introduced into the solution at 301 s, the dissolved H_2_ drops sharply, while the dissolved O_2_ rises from 2.7 mg/L to 19.9 mg/L. This implies that the driving reaction of the Mg-based micromotors is transformed to Pt-catalyzed H_2_O_2_ decomposition (producing O_2_) instead of the Mg-H_2_O reaction (producing H_2_). The initial O_2_ concentration in the aqueous solution stems from dissolved air. This transformation arises from the strong oxidization of H_2_O_2_ with a much higher electrode potential than that of H_2_O [30]. In the presence of H_2_O_2_, Mg prefers to react with H_2_O_2_ without generating H_2_ rather than to react with H_2_O [54] and simultaneously Pt-catalyzed H_2_O_2_ decomposition can produce O_2_ gas. This can be further verified by the fact that no bubble generates when bare Mg microparticles are put in H_2_O_2_ aqueous solution (Video S3).

**Figure 4:**
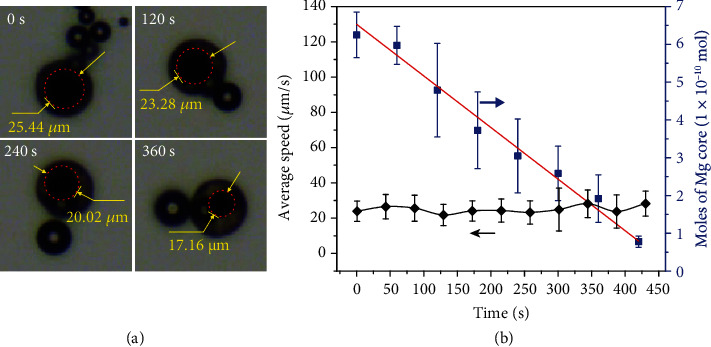
The Mg-based micromotor propelled by the Mg-H_2_O reaction. (a) Time-lapse microscope images of Mg core consumption in the solution without H_2_O_2_. (b) The corresponding average speeds in every 40 s of the Mg-based micromotor and Mg core amounts versus time. The red line indicates the fitting line for Mg core consumption.

As discussed above, the Mg-based micromotor can be driven by both Mg-H_2_O reaction in the aqueous solution without H_2_O_2_ and Pt-catalyzed H_2_O_2_ decomposition when meeting H_2_O_2_. In the aqueous solution containing NaHCO_3_ and PVP but without H_2_O_2_, the Mg core is consumed continuously via the Mg-H_2_O reaction to produce numerous H_2_ bubbles that propel the micromotor due to the conservation of momentum [55].  Figure 4 shows the Mg core amounts and motion speeds versus time, respectively. After overcoming the initial gravity-induced subsidence (Figure S4 and Video S4), the micromotor keeps moving by bubble recoils with the Mg core amount continuously decreasing as shown in Figure 4(a) and Video S5, indicating its self-consumed feature. At this time, the lifetime of the micromotor mainly depends on the opening size (Figure S5) and is in the range of a few minutes to dozens of minutes. With the Mg-H_2_O reaction proceeding, there forms a confined space inside the micromotor, where the nucleation and growth of H_2_ bubbles occur. At the early stages, the ejected bubbles are small, while they become large when the Mg core is almost gone. The size of the formed bubbles is mainly dependent on the growth time in the confined space. At the initial stages, the narrow space between the Mg core and the coating layer is too narrow for the growth of bubbles before they eject from the micromotor due to the capillary effect. Upon the enlarged confined space over time, the growth period is extended resulting in the formation of large bubbles.  Figure 4(b) shows that the as-prepared micromotor has a relatively constant motion speed and consumption rate of the Mg core during Mg-H_2_O reaction. This is not in agreement with the previous reported surface area-related reaction rate and can be explained by the effect of the confined space, in which the permeation of water is competitive with the escape of bubbles.

**Figure 5:**
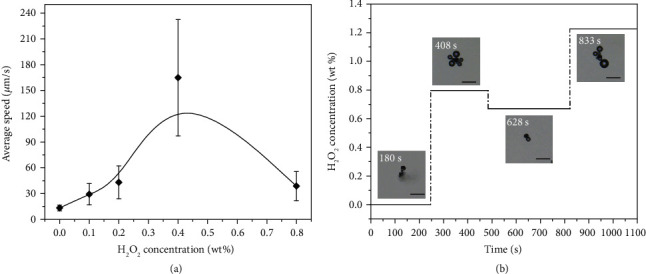
The effect of H_2_O_2_ concentration on motion behaviors of the Mg-based micromotor. (a) The average speed of the Mg-based micromotor in a solution with different concentrations of H_2_O_2_ at 38°C. The black line represents the trend line. (b) The H_2_O_2_ concentration of the aqueous solution containing the Mg-based micromotor versus time. The insets show typical states of the micromotor in each period, and the dotted line represents a transitional period after which a constant local H_2_O_2_ concentration is available in the system. Scale bar: 100 *μ*m. In the aqueous solution without H_2_O_2_ (0~250 s), bubbles generate from the open end of the Mg-based micromotor, while adding H_2_O_2_ to raise the concentration to 0.8 wt% results in bilateral bubble production (250~480 s). Further reducing H_2_O_2_ concentration to 0.67 wt% via diluting makes the reaction occur in a unilateral side (480~820 s), and the reaction again occurs in bilateral sides when the concentration reaches 1.23 wt% by adding H_2_O_2_ again (820~1100 s).

The as-constructed Mg-based micromotors can improve the motion controllability and lifetime by the spontaneous transformation of propulsion mechanisms from the self-consuming reaction to the catalytic reaction in terms of the surroundings. In H_2_O_2_ aqueous solution, the as-prepared Mg-based micromotor can be driven by Pt-catalyzed H_2_O_2_ decomposition and keeps constantly moving until the fuel (H_2_O_2_) runs out. The lifetime of the motor moving in H_2_O_2_ aqueous solution is determined by the amount of H_2_O_2_ and has been significantly prolonged because of the far excess “fuel” in the system.  Figure 5 shows the effect of H_2_O_2_ concentration on the motion behaviors of the Mg-based micromotors. Taking a micromotor moving at 38°C for example, the as-prepared micromotor first accelerates and then decelerates the motion with increasing the H_2_O_2_ concentration (Figure 5(a)). This is dramatically different from the previously reported catalytic micromotors, whose speeds generally increase with increasing catalytic substrate concentrations [56–58]. The speeds of the Mg-based micromotor before and after the addition of H_2_O_2_ with different concentrations are shown in Figure S6. When the micromotor moves in the solution without H_2_O_2_, the motion speed is basically stable with an average value of about 13.2 *μ*m/s depending on the relatively constant H_2_ bubble size and generation frequency (Video S5). After adding H_2_O_2_, the motion speeds of the micromotor contributed by O_2_ detachment from Pt-catalyzed H_2_O_2_ decomposition are closely related to H_2_O_2_ concentration. The initial increase of the speed with increasing H_2_O_2_ concentration (0~0.4 wt%) is attributed to an increased bubble generation frequency. The large variability of speed in 0.4 wt% H_2_O_2_ aqueous solution may be attributed to the rapid changes of *α* and the bubble escape positions in motion (Video S6). The remarkable decrease of motion speed occurs as the H_2_O_2_ concentration is further increased to 0.8 wt%, implying a possible change of motion behavior. As shown in Figure 5(b) and Video S7, the micromotor displays a self-limitation motion like hovering due to the bilateral O_2_ bubble generation and release as H_2_O_2_ concentration is higher than 0.8 wt%. This indirectly reduces the asymmetry of the micromotor and leads to the reduction of the speed. After diluting 0.8 wt% H_2_O_2_ aqueous solution to 0.67 wt%, the micromotor shows a curved ballistic trajectory as the O_2_ bubbles are released from the opening instead of bilateral release, while the bilateral O_2_ bubble generation and detachment occur again after supplementing H_2_O_2_ to 1.23 wt%. In other words, the as-prepared micromotor exhibits a self-limitation behavior in a relatively high-concentration H_2_O_2_ aqueous solution and returns to a curved ballistic motion at a low concentration. The transformation is reversible with changing the concentration of H_2_O_2_. Such unique concentration-induced self-limitation is associated with the permeability of H_2_O_2_ into the PNIPAM hydrogel layer. A high concentration of H_2_O_2_ aqueous solution can form a large concentration gradient between the two sides of the hydrogel layer to facilitate more H_2_O_2_ across the PNIPAM hydrogel layer and react with the middle Pt layer. As a result, the generated O_2_ accumulates and reaches the heterogeneous nucleation energy on the side of the hydrogel layer for O_2_ bubble nucleation and growth, resulting in a concentration-induced self-limitation.

**Figure 6:**
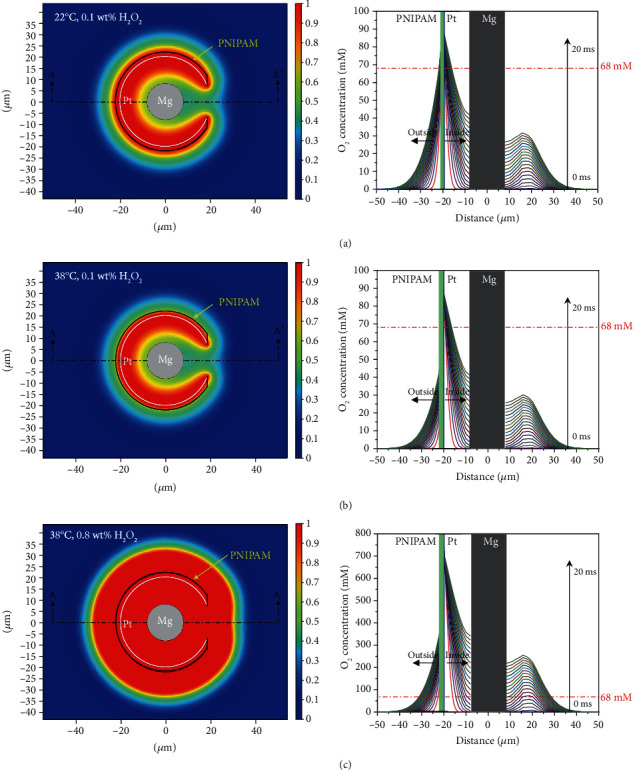
The normalized O_2_ concentration distribution around the Mg-based micromotor at (a) 22°C in 0.1 wt% H_2_O_2_, (b) 38°C in 0.1 wt% H_2_O_2_, and (c) 38°C in 0.8 wt% H_2_O_2_ aqueous solution. The right panels show the curves of theoretical O_2_ concentration on AA′ line segments within 20 ms versus the distance apart from the center of Mg core at different time intervals. The critical nucleation concentration of O_2_ bubble is 68 mM.

To further illustrate the temperature/concentration-dependent self-limitation motion mechanisms of the Mg-based micromotor driven by Pt-catalyzed H_2_O_2_ decomposition, we have carried out numerical simulations over the Mg-based micromotor based on a 2D model (Figure S7) by chemical reaction engineering and dilute chemical species transport modules of COMSOL Multiphysics.  Figure 6 shows the numerical simulation results of O_2_ concentration distribution around the Mg-based micromotor with different temperatures and H_2_O_2_ concentrations. It should be noted that the simplified model only considers the O_2_ concentration distribution for nucleation and ignores the fluid disturbance deriving from micromotor motion. In this model, the PNIPAM coating is treated as a porous media, because the swelling/shrinking of the hydrogel network can result in different porosities for O_2_ diffusion, which is temperature-dependent. In a swelling state, a large cooperative diffusion coefficient benefits the permeation of H_2_O_2_ through the hydrogel layer resulting in a network with large porosity, which is approximately twice than that in a shrinking state [44, 59]. When the micromotor is moving below the LCST at a low-concentration H_2_O_2_ aqueous solution of 0.1 wt%, the O_2_ generated inside the coating layer will reach the critical nucleation concentration of 68 mM within 1 ms due to the accumulative effect of the inner confined void (Figure 6(a)). Simultaneously, in view of the water-swelling feature of the PNIPAM hydrogel layer at this temperature, there are more H_2_O_2_ permeating across the hydrogel layer to react with the middle Pt layer resulting in O_2_ nucleation on the side of the hydrogel layer. As a result, the micromotor shows a self-limitation behavior caused by bilateral O_2_ bubble generation (Figure 2(a), iv). In contrast, by increasing temperature to 38°C, the access of H_2_O_2_ is slowed down by the contracted PNIPAM hydrogel layer. Consequently, the concentration of O_2_ generated on the side of the hydrogel layer cannot reach its critical nucleation concentration under continuous outward diffusion (Figure 6(b)). Therefore, the inside generated O_2_ bubble detachment from the opening is the origin of the sufficient propulsion of the as-prepared Mg-based micromotor (Figure 2(a), ii). However, increasing the H_2_O_2_ concentration to 0.8 wt% could facilitate the permeation of H_2_O_2_ aqueous solution into the PNIPAM hydrogel layer and the catalytic decomposition of H_2_O_2_ at the middle layer Pt (Figure 6(a)). This enables the O_2_ generation on both sides of the middle Pt layer to reach the critical nucleation concentrations and thus induce self-limitation motion of the micromotor.

## 3. Conclusion

In this work, we present a kind of dual mechanism-propelled Mg-based micromotor with motion responsive to both temperature and H_2_O_2_ concentration. The as-constructed Mg-based micromotors are obtained by asymmetrically coating of Mg microspheres with catalytically active Pt and temperature-sensitive PNIPAM hydrogel layers in sequence. They can be propelled by Mg-H_2_O reactions followed by Pt-catalyzed H_2_O_2_ decomposition when encountering H_2_O_2_ and exhibit extended motion lifetime. In the latter case, they would respond to both H_2_O_2_ concentration and temperature in solution environment, demonstrating unique temperature- and/or concentration-induced self-limitation motion like hovering. This is associated with the temperature-dependent diffusion coefficient and the concentration-related permeability of H_2_O_2_ aqueous solution in the PNIPAM hydrogel layer. Our results have for the first time demonstrated the Mg-based micromotors with motion responsive to external stimuli in a multifuel system and provided a strategy to push forward the development of intelligent Mg-based micromotors and to prolong the motion lifetime.

## 4. Materials and Methods

### 4.1. Materials

All the chemicals used in this work were of analytical grade and were used as received without further purification. Poly(vinylpyrrolidone) (PVP, K30), ethylene glycol (EG), hydrogen peroxide, N-isopropylacrylamide (NIPAM), bis(N,N-methylene bis(acrylamide)), 2,2-diethoxyacetophenone (DEAP), sodium bicarbonate (NaHCO_3_), acetone, and ethanol were purchased from TCI (Shanghai) Development Co., Ltd., China. Commercial Mg microspheres were purchased from TangShan WeiHao Magnesium Powder Co. (Tangshan, China) and washed with acetone twice before usage.

### 4.2. Fabrication of the Mg-Based Micromotors

The Mg-based micromotors are prepared by our previously reported method with some modifications [29] shown in Figure S1. In detail, Mg microspheres with an average size of about 40 *μ*m were completely scattered on the surface of a glass slide precoated with a thin poly(vinylpyrrolidone) (PVP, K30) film by dripping 100 *μ*L 0.5 wt% PVP ethanol solution on the glass slide and then drying at 60°C for 10 min. Then, the PVP-glass slide with dispersed Mg microspheres was placed in humid air with a relative humidity of 50% for 20 s. During this process, Mg microspheres were partially immersed in the PVP film under the gravitational field to obtain partially covered carriers. After drying in an oven at 60°C for 10 min, the Mg microspheres with partially covered bottom surface were fixed in the PVP film. Then, the exposed Mg surfaces were coated with a Pt layer via magnetron sputtering for 240 s to obtain the Mg/Pt microspheres fixed. The poly(N-isopropylacrylamide) (PNIPAM) precursor solution was prepared by dissolving 0.35 g NIPAM in 3.5 mL EG followed by adding 0.2 g bis(N,N-methylene bis(acrylamide)) and 20 *μ*L 2,2-DEAP into the solution. Subsequently, 100 *μ*L PNIPAM-EG precursor solution was dropped onto the obtained glass slide to form a liquid film on the surface of the Mg/Pt microspheres fixed on the PVP-glass slide followed by spin-coating by a spin coater (speed of 2000 rpm, coating time of 50 s, EZ4 spin coater, Schwan Technology) and then covered by a slip. The obtained glass slide was exposed to the UV light to polymerize NIPAM for 240 s followed with removing the thin cover slips and drying in vacuum at 60°C for 12 h. Finally, the Mg-based micromotors were separated from the substrate by a blade-scratching process and washed with acetone twice to remove the PVP layer.

### 4.3. Motion Tests of the Mg-Based Micromotors

The movement of the as-prepared Mg-based micromotors was performed in 0.5 M NaHCO_3_ aqueous solution with 5 wt% PVP as surfactant. In each test, 90 *μ*L 0.5 M NaHCO_3_ and 5 wt% PVP containing the micromotors were dispersed on a glass slide. The glass slide was heated from room temperature to the desired temperature using the microscope temperature control stage. Then, 10 *μ*L H_2_O_2_ aqueous solution with different concentrations was dropped on the glass slide for observation and recorded with an optical microscope using a high-resolution CCD digital camera (Leica DM 3000B, Inc. Germany).

### 4.4. Determination of Dissolved H_2_ and O_2_

To measure dissolved O_2_ and dissolved H_2_ in the system, 20 mg Mg-based micromotors was added into 90 mL 0.5 M NaHCO_3_ and 5 wt% PVP solution at 22°C. After 301 s, 10 mL 1 wt% H_2_O_2_ was added into the system. During the whole process, the data was measured and recorded by the portable dissolved H_2_ and O_2_ analyzers.

### 4.5. Characterizations

Scanning electron microscopy (SEM) images were taken with a Hitachi S-4800 (Japan) at an acceleration voltage of 10.0 kV. The energy dispersive spectroscopy (EDS) analyses were carried out on a JEOL JEM-7500F (Tokyo, Japan) operated at an accelerating voltage of 15 kV. Magnetron Sputtering System (JCP500, Beijing Technol Science Co., Ltd., China) was used to cover the middle Pt layer. The microscopy images and the motion videos of micromotors were recorded with optical microscope using a high-resolution CCD digital camera (Leica DM 3000B, Inc. Germany) coupled with ×5, ×10, ×20, and ×40 objective. The temperature was heated by heating accessories (TempController 2000-1, Inc. Germany) of the microscope. All videos were analyzed by Video Spot Tracker V08.01 and Matlab R2018a software.

### 4.6. Numerical Simulation

In order to further confirm the role of temperature and H_2_O_2_ concentration in the transformation of the limitation behavior, a two-dimensional numerical simulation of the process is performed using a commercial COMSOL finite element analysis software. The chemical reaction engineering and dilute chemical species transport module are employed. Porous media transfer is used based on the effect of temperature on the PNIPAM hydrogel to simulate the response. The two-dimensional model is created according to the state of the motor at 360 s in Figure 4(a) with an approximately 50 nm Pt layer and 2 *μ*m PNIPAM layer as shown in Figure S7. The reaction engineering module assumes a batch type reactor process with constant volume. The decomposition reaction is

(2) $$\begin{array}{c} 2{\text{H}}_{2}{\text{O}}_{2}\overset{\text{Pt}}{⟶}2{\text{H}}_{2}\text{O}+{\text{O}}_{2}↑ \end{array}$$

The reaction rate constant *k*_*f*_ changing with temperature according to Arrhenius expression is

(3) $$\begin{array}{c} k_{f}=A_{f}*\exp\left(\frac{-E_{a}}{RT}\right). \end{array}$$

The values for activation energy *E*_*a*_ are given as 40 kJ/mol, frequency factor of collision *A*_*f*_ as 4.32 × 10^5^ min^−1^, and reaction rate constant *k*_*f*_ as 3.60 × 10^−2^ min^−1^ (for 22°C) and 8.32 × 10^−2^ min^−1^ (for 38°C). The surface species (catalyst) is kept with locked concentration. The governing equations in reaction engineering are

(4) $$\begin{array}{c} \frac{dc_{i}}{dt}=R_{i}+R_{\text{ads},i}\frac{A_{r}}{V_{r}}. \end{array}$$

Here, for a species *i*, *c*_*i*_ is the concentration, *R*_*i*_ is the rate of the species, *R*_ads,*i*_ is the rate effect of surface species on *i*, *A*_*r*_ is the surface reaction area, and *V*_*r*_ is the reactor volume. The study is carried out with time steps of 1 to 20 ms. Transport of diluted species is used to compute the concentration field. The governing physics involves the use of Fick's law for transport by diffusion and adsorption as the adsorption happens on the catalytic surface. The products are formed when they are desorbed from the catalyst surface. And simplify the PNIPAM hydrogel into a porous medium. The governing equation for this problem is the conservative form of the transport of diluted species in porous media interface:

(5) $$\begin{array}{c} \frac{d\left(\varepsilon_{p}c_{i}\right)}{dt}+\nabla ∙J_{i}=R_{i},\\ J_{i}=-D_{e,i}\nabla c_{i},\\ D_{e,i}=\frac{\varepsilon_{p}}{\tau_{F,i}}D_{F,i}. \end{array}$$

Here, for a species *i*, **J**_*i*_ is the flux of the species, *ε*_*p*_ is the porosity of the hydrogel, *τ*_*F*,*i*_ is the tortuosity factor based on the Millington-Quirk model [60], *D*_*F*,*i*_ is the diffusion coefficient of the species in fluid, and *D*_*e*,*i*_ is the effective diffusion coefficient of the species. The typical simulation parameters are shown in Table S1.

## Supplementary Materials

Supplementary 1 Figure S1: fabrication process of the Mg-based micromotors. Figure S2: SEM and EDS mapping images of a typical Mg-based micromotor. Figure S3: time-lapse microscope images of a typical Mg-based micromotor showing self-limitation with a small angular velocity (3.02°/s). Figure S4: initial gravity-induced subsidence of a typical Mg-based micromotor. Figure S5: the curve of the lifetime of a typical Mg-based micromotor versus the average opening size. Figure S6: instantaneous speed of the Mg-based micromotor before and after adding H_2_O_2_ with different concentrations. Figure S7: schematic of the two-dimensional model for the Mg-based micromotor. Table S1: typical parameters used in the simulations.

Supplementary 2 Supporting Video S1: a typical Mg-based micromotor moving without H_2_O_2_ (i and iii) or with 0.1 wt% H_2_O_2_ aqueous solution (ii and iv) at 38°C (i and ii) or 22°C (iii and iv), respectively.

Supplementary 3 Supporting Video S2: a typical Mg-based micromotor showing self-limitation with a small angular velocity (3.02°/s).

Supplementary 4 Supporting Video S3: the states of a typical bare Mg microparticle in the aqueous solution with or without H_2_O_2_.

Supplementary 5 Supporting Video S4: initial gravity-induced subsidence of a typical Mg-based micromotor.

Supplementary 6 Supporting Video S5: Mg core consumption during the Mg-H_2_O reaction.

Supplementary 7 Supporting Video S6: a typical Mg-based micromotor moving in 0.4 wt% H_2_O_2_ aqueous solution.

Supplementary 8 Supporting Video S7: H_2_O_2_ concentration-dependent motion behaviors of a typical Mg-based micromotor.
