# Supplementary material for: Mg-Based Micromotors with Motion Responsive to Dual Stimuli
Source: Research (Wash D C). 2020 Aug 4;2020:6213981. doi: 10.34133/2020/6213981 (PMC7424550; doi:10.34133/2020/6213981)
Supplement: Supplementary 1 — Figure S1: fabrication process of the Mg-based micromotors. Figure S2: SEM and EDS mapping images of a typical Mg-based micromotor. Figure S3: time-lapse microscope images of a typical Mg-based micromotor showing self-limitation with a small angular velocity (3.02°/s). Figure S4: initial gravity-induced subsidence of a typical Mg-based micromotor. Figure S5: the curve of the lifetime of a typical Mg-based micromotor versus the average opening size. Figure S6: instantaneous speed of the Mg-based micromotor before and after adding H2O2 with different concentrations. Figure S7: schematic of the two-dimensional model for the Mg-based micromotor. Table S1: typical parameters used in the simulations. [file 6213981.f1.docx]

**SUPPORTING INFORMATION**

**Mg-based Micromotors with Motion Responsive to Dual Stimuli**

*Kang Xiong, Leilei Xu,* Jinwei Lin, Fangzhi Mou, Jianguo Guan**

State Key Laboratory of Advanced Technology for Materials Synthesis and Processing, International School of Materials Science and Engineering, Wuhan University of Technology, Wuhan 430070, P. R. China.

* Corresponding authors.

Email: [xull@whut.edu.cn](mailto:xull@whut.edu.cn); guanjg@whut.edu.cn

**Supporting Figures**


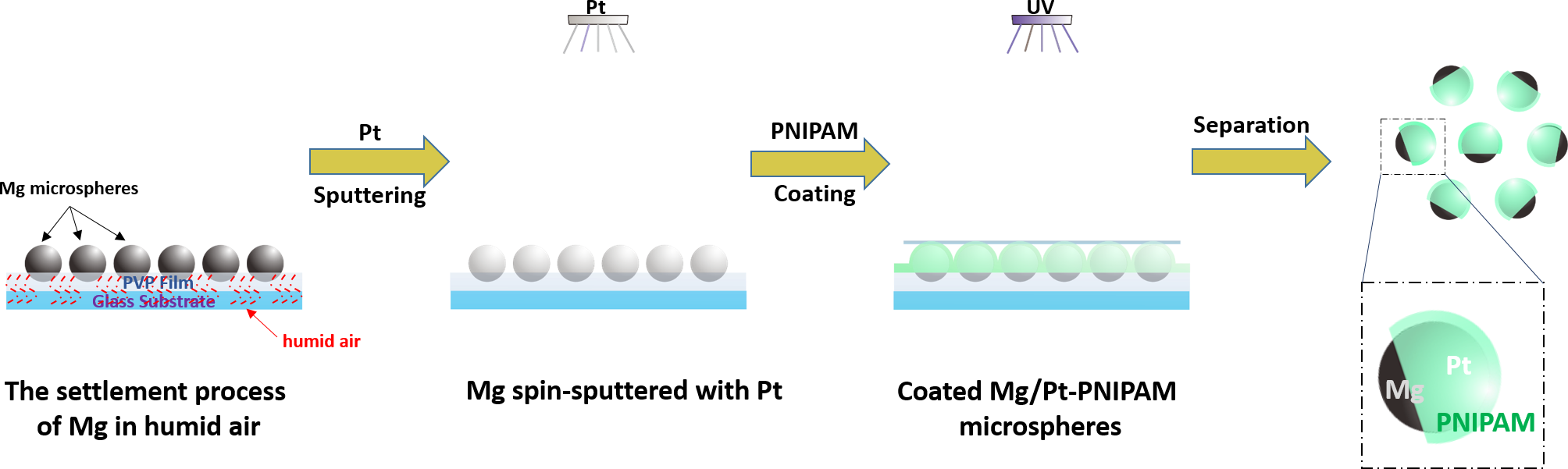


**Fig. S1. Fabrication process of the Mg-based micromotors.** A monolayer of Mg microspheres are positioned on a glass slide with PVP film during a settlement process and the uncovered area is subsequently spin-sputtered with a Pt layer and coated by PNIPAM hydrogel via magnetron sputtering and UV polymerization techniques, respectively.


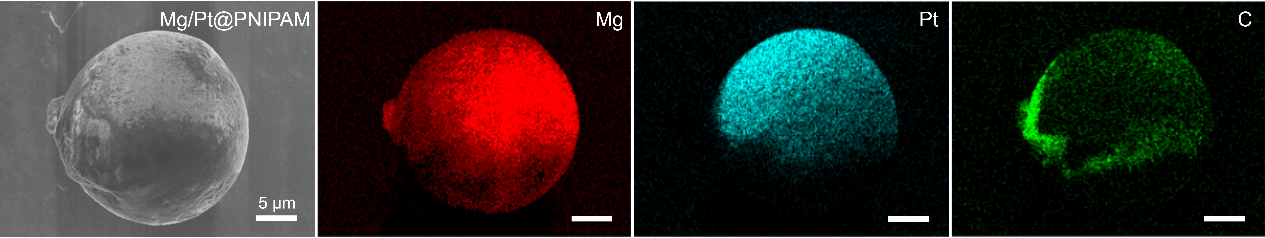


**Fig. S2. SEM and EDS mapping images of a typical Mg-based micromotor.**


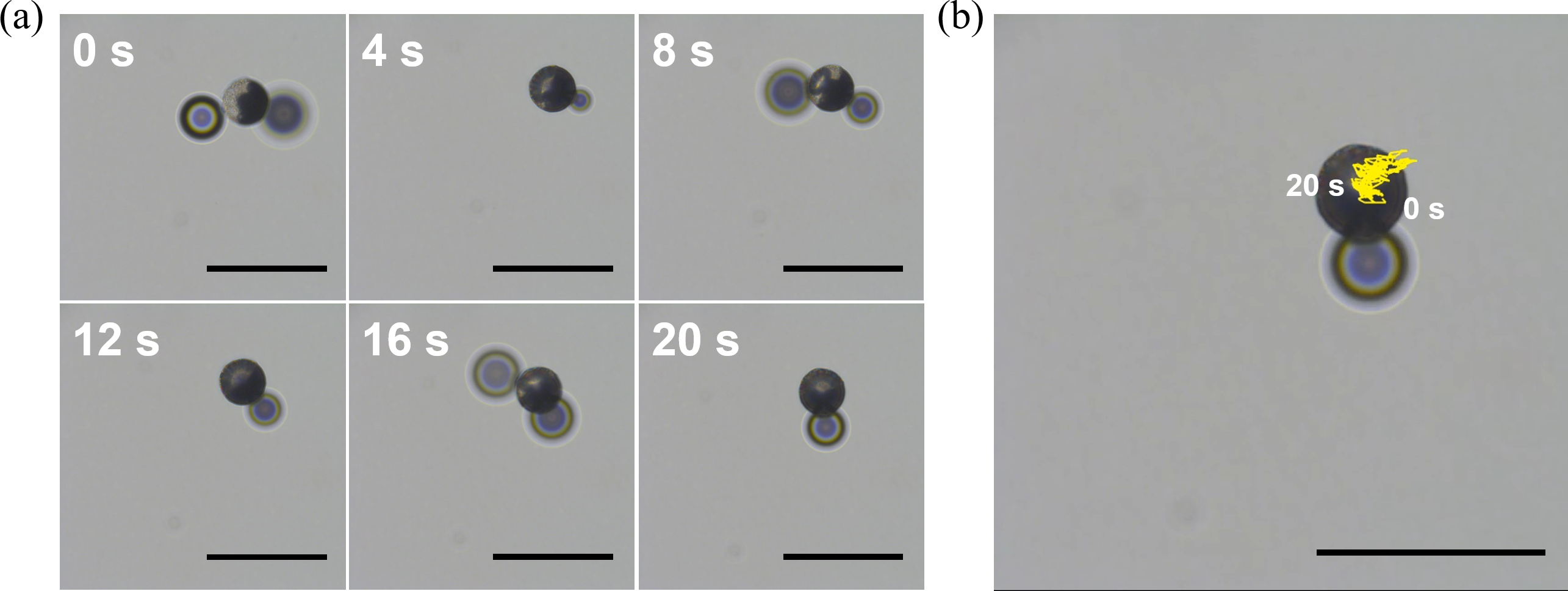


**Fig. S3. Time-lapse microscope images a typical Mg-based micromotor showing self-limitation with a small angular velocity (3.02 °/s).** (a) Time-lapse images of a typical Mg-based micromotor with a small translation speed and rotation speed during 20 s. (b) The corresponding motion trajectory image with a small displacement (18.16 μm). Scale bar: 100 μm.


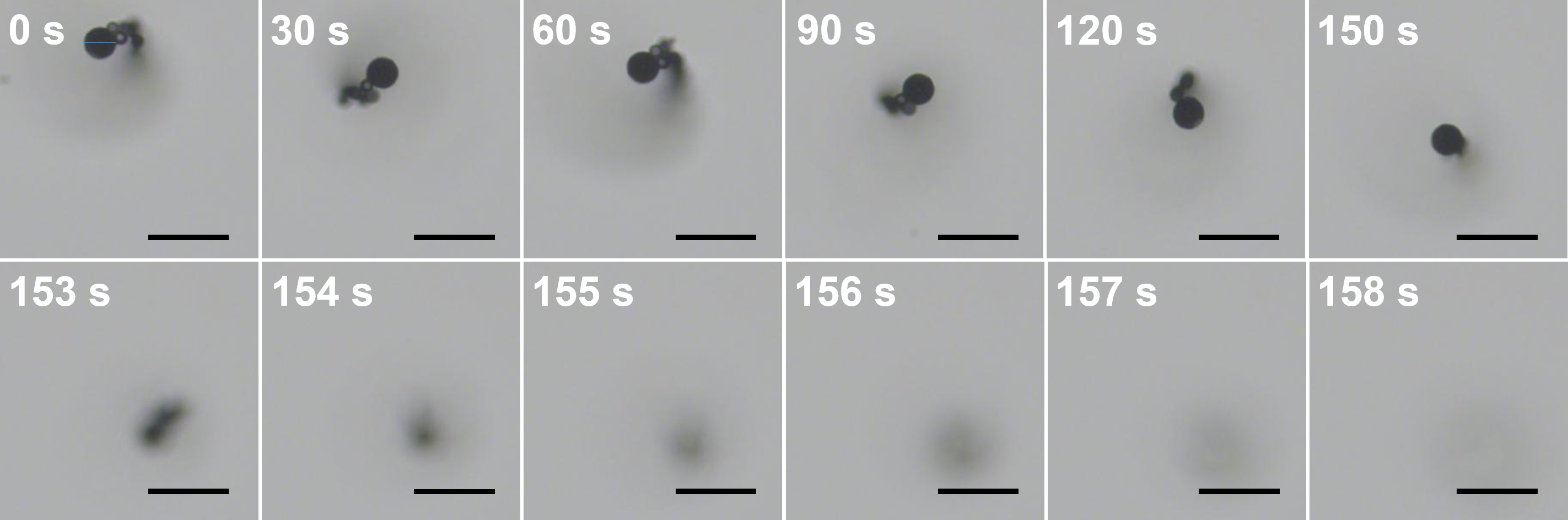


**Fig. S4**. **Initial gravity-induced subsidence of a typical Mg-based micromotor.** Time-lapse microscope images showing a typical Mg-based micromotor detached from the substrate after a period of Mg-H_2_O reaction in 0.5 M NaHCO_3_ + 5 wt% PVP solution. Scale bar: 100 μm.

**
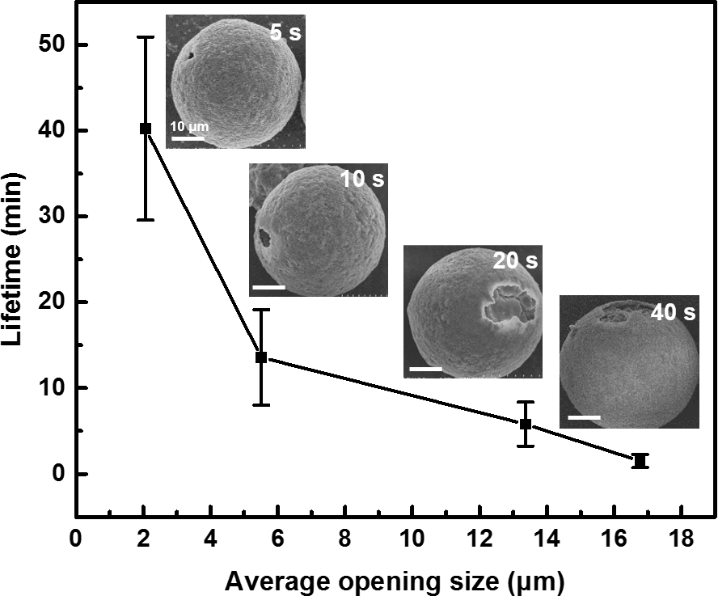
**

**Fig. S5. The curve of the lifetime of a typical Mg-based micromotor versus the average opening size. The insets represent the SEM images of the Mg-based micromotor setting on different times.**


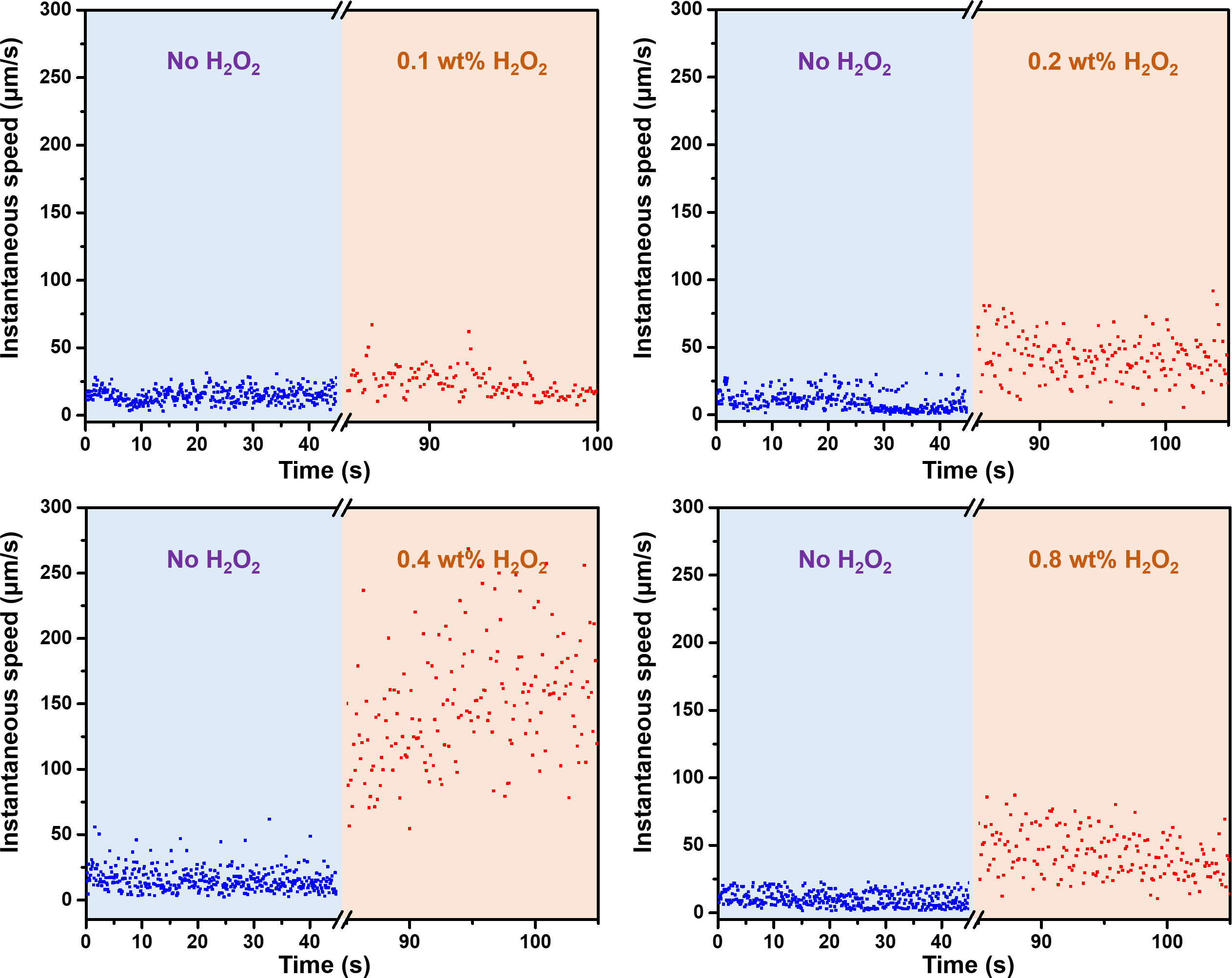


**Fig. S6**. **Instantaneous speed of the Mg-based micromotor before and after adding H_2_O_2_ with different concentrations.**

**
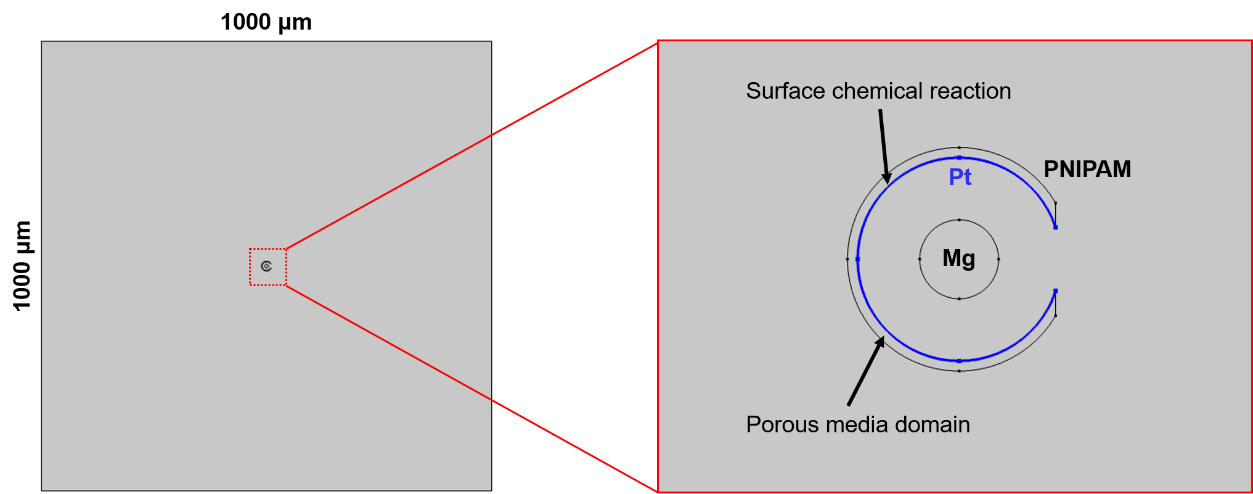
**

**Fig. S7. Schematic of the two-dimensional model for the Mg-based micromotor.**

**Table S1.** **Typical parameters used in the simulations.**

| Parameter | Meaning | Value |
| --- | --- | --- |
| S_Pt | **Pt catalyst surface molar concentration** | **4.38 x 10^-3^ [mol/m^2^]** |
| L_Pt | **Pt layer thickness** | **50[nm]** |
| Cm_O_2_ | **Maximum supersaturation concentration of O_2_** | **68 [mM]** |
| D_H_2_O_2_ | **H_2_O_2_ diffusion coefficient** | **6.6 x 10^-10^ [m^2^/s]** |
| D_H_2_O | **H_2_O diffusion coefficient** | **6.6 x 10^-10^ [m^2^/s]** |
| D_O_2_ | **O_2_ diffusion coefficient** | **2 x 10^-9^ [m^2^/s]** |
| R_Mg | **Mg core radius** | **8.58 [μm]** |
| Epsilon_p | **Porosity ratio of the PNIPAM hydrogels at 25 °C and 35 °C** | **0.5** |

**Supporting Videos**

**Video S1.** The Mg-based micromotor moving in the aqueous solution without H_2_O_2_ (i and iii) or with 0.1 wt% H_2_O_2_ (ii and iv) at 38 ^o^C (i and ii) or 22 ^o^C (iii and iv), respectively.

**Video S2.** A typical Mg-based micromotor showing self-limitation with a small angular velocity (3.02 °/s).

**Video S3.** The states of a typical bare Mg microparticle in the aqueous solution with or without H_2_O_2_.

**Video S4.** Initial gravity-induced subsidence of a typical Mg-based micromotor.

**Video S5.** Mg core consumption during the Mg-H_2_O reaction.

**Video S6.** A typical Mg-based micromotor moving in 0.4 wt% H_2_O_2_ aqueous solution.

**Video S7.** H_2_O_2_ concentration-dependent motion behaviors of a typical Mg-based micromotor.
